# Hypoxic areas, density-dependence and food limitation drive the body condition of a heavily exploited marine fish predator

**DOI:** 10.1098/rsos.160416

**Published:** 2016-10-26

**Authors:** Michele Casini, Filip Käll, Martin Hansson, Maris Plikshs, Tatjana Baranova, Olle Karlsson, Karl Lundström, Stefan Neuenfeldt, Anna Gårdmark, Joakim Hjelm

**Affiliations:** 1 Department of Aquatic Resources, Institute of Marine Research, Swedish University of Agricultural Sciences, Turistgatan 5, 45330 Lysekil, Sweden; 2 Swedish Meteorological and Hydrological Institute, Sven Källfelts gatan 15, 42671 Gothenburg, Sweden; 3 Fish Resources Research Department, Institute of Food Safety, Animal Health and Environment, Daugavgrivas 8, 1048 Riga, Latvia; 4 Department of Environmental Research and Monitoring, Swedish Museum of Natural History, Box 50007, 10405 Stockholm, Sweden; 5 Department of Aquatic Resources, Swedish University of Agricultural Sciences, Institute of Coastal Research, Skolgatan 6, 74242 Öregrund, Sweden; 6 National Institute of Aquatic Resources, Technical University of Denmark, 2920 Charlottenlund, Denmark

**Keywords:** cod (*Gadus morhua*), hypoxia, suitable habitat, density-dependence, prey availability, Baltic Sea

## Abstract

Investigating the factors regulating fish condition is crucial in ecology and the management of exploited fish populations. The body condition of cod (*Gadus morhua*) in the Baltic Sea has dramatically decreased during the past two decades, with large implications for the fishery relying on this resource. Here, we statistically investigated the potential drivers of the Baltic cod condition during the past 40 years using newly compiled fishery-independent biological data and hydrological observations. We evidenced a combination of different factors operating before and after the ecological regime shift that occurred in the Baltic Sea in the early 1990s. The changes in cod condition related to feeding opportunities, driven either by density-dependence or food limitation, along the whole period investigated and to the fivefold increase in the extent of hypoxic areas in the most recent 20 years. Hypoxic areas can act on cod condition through different mechanisms related directly to species physiology, or indirectly to behaviour and trophic interactions. Our analyses found statistical evidence for an effect of the hypoxia-induced habitat compression on cod condition possibly operating via crowding and density-dependent processes. These results furnish novel insights into the population dynamics of Baltic Sea cod that can aid the management of this currently threatened population.

## 1. Introduction

Fish body condition is a key parameter in the dynamics of fish populations. Variations of condition in wild fish populations have been attributed to several factors, such as food availability [1], density-dependence [2], hydrological circumstances such as temperature [3], parasitic infection [4] or selective fishing [5]. It has been shown that fish condition can affect fish natural mortality [6] and reproductive potential [7] and, therefore, it is considered to play a crucial role in shaping the dynamics of fish populations [8,9].

In the Central Baltic Sea, a large focus has been addressed to investigate the factors driving the body condition of pelagic clupeid species, i.e. herring *Clupea harengus* and sprat *Sprattus sprattus* [10–14]. On the other hand, less emphasis has been given to the condition of cod (*Gadus morhua*), the main piscivore and the most commercially important species of the system [15,16]. The Eastern Baltic cod population (hereafter simply referred to as Baltic cod) has undergone dramatic abundance changes in the past four decades, as a result of fishing pressure and hydro-climatic variations [17]. Concomitant with these changes, large variations in mean body condition, but also size, have been observed, with a substantial decrease during the past 20 years [17,18]. The low mean body condition and size of cod in the Baltic Sea has been also emphasized by the fishing industry that lamented catches rich in lean and undersized specimens, with detrimental effects on the catch values and thus revenues [16]. Low condition has also been suggested as one of the causes of the recent disappearance of large cod individuals, via increased mortality or decreased growth [19]. Analyses performed on long time-series of stomach contents evidenced a decrease in prey weight in the cod stomachs during the past 20 years, which suggests a reduction in feeding rate as one of the main causes of the observed decline in condition [20]. However, despite the large changes in condition and the consequences for the fishery and the ecosystem, the original causes of these changes are still elusive. In the literature, cod condition has been correlated to prey availability [21], but there is a lack of studies accounting for the possible simultaneous effect of multiple factors. Especially, the potential direct and indirect effects of abiotic variability on cod condition have been neglected in previous studies.

One of the most prominent abiotic changes that occurred in the Baltic Sea ecosystem during the past two decades has been the fivefold increase in the extent of hypoxic and anoxic areas [22,23], which is part of a global-scale phenomenon [24]. The expansion of hypoxic and anoxic areas (often referred to as ‘dead zones’) can have multiple direct and indirect effects on aquatic organisms and entire ecosystems [24–26], as shown in marine, brackish and freshwater habitats [27–29]. Especially, studies undertaken both in the wild and within experiment set-ups have shown the large effects of hypoxia on basic metabolism, ecology and life-history traits of fish, including growth and condition [30–33].

Another factor that previously has been related to Baltic cod condition is the intensity of infection with the parasites *Pseudoterranova decipiens* (cod worm) and *Contracaecum osculatum* (liver worm) [34]. The infection intensity has increased in Baltic Sea cod during the past 30 years, probably due to the increase of the population of grey seals (*Halichoerus grypus*) [34–36], that are the final host of these parasites.

Here we used for the first time a newly compiled time-series of fishery-independent biological data to investigate the changes of Baltic cod condition during the past 40 years. We firstly characterized the variations in condition by investigating the potential differences between areas and size-classes. Thereafter, the changes in condition were put in relation to both the biological context (cod abundance, prey availability and seal abundance) and hydrological circumstances (extent of hypoxic areas). These analyses, by discerning the main factors relating to cod condition, are essentials for the management of this ecologically and commercially key species in the Baltic Sea.

## 2. Material and methods

**Figure 1.**
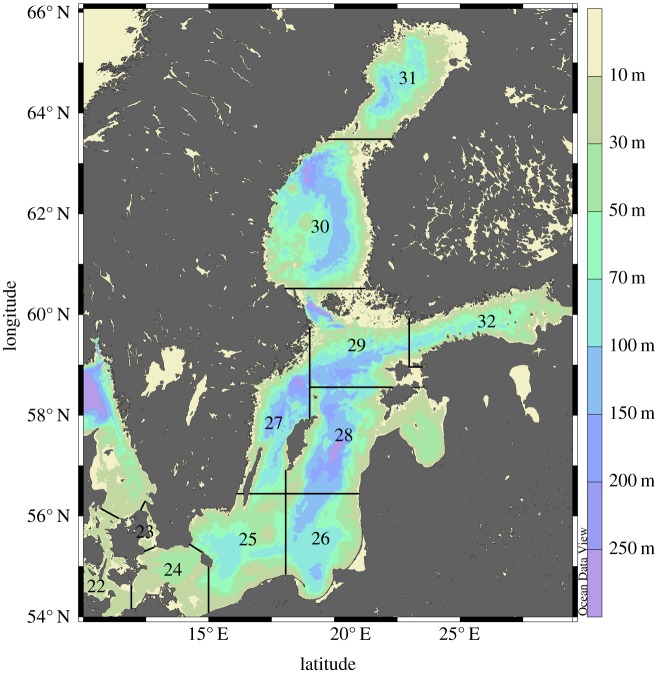
Map of the Baltic Sea. The study area includes the ICES subdivisions (SDs) 25–28 (i.e. the Central Baltic Sea).

Biological data on Eastern Baltic cod individuals were collected during the Baltic International Trawl Survey (BITS, [37]) between 1991 and 2014 in the International Council for the Exploration of the Sea (ICES) subdivisions (SDs) 25–28 (figure 1). The data include individual fish total length (*L*), total weight (*W*), age, sex and maturity stage and were retrieved from the ICES DATRAS database (www.ices.dk). Further data collected during bottom trawl surveys performed in 1976–1990 were retrieved from the national databases of the former Swedish Board of Fisheries (currently the Department of Aquatic Resources, Swedish University of Agricultural Sciences) and the former Baltic Research Institute of Fishery of Latvia (currently the Latvian Institute of Food Safety BIOR). Cod individual body condition (Fulton's *K*) was estimated as *K* = *W*/*L*^3^ × 100, where *W* is the total weight (g) and *L* the total length (cm) of the fish. Condition was then averaged per 10 cm length-class (10–19 cm, 20–29 cm, 30–39 cm, 40–49 cm, 50–59 cm) for each SD, year and country. Thereafter, a generalized linear model (GLZM) was used to predict the year effect on cod condition for each SD after scaling out the country effect. Typically, during the surveys each country covers a specific area within the same SD. Therefore, this correction allowed accounting for potential spatial differences in condition within an SD when its area was not entirely covered by the survey. Condition data were normally distributed, and thus the normal distribution with an identity function was used in the GLZMs. This procedure has been used before to estimate SD-specific condition of Baltic Sea sprat [14]. Mean condition for the whole Central Baltic Sea (SDs 25–28) was then estimated by averaging the SD-specific estimates because no difference was found between SDs (see Results). Lengths < 10 cm and ≥60 cm were excluded because they were not well represented in the data (*n* < 50 for some SDs, countries or years). We focused on the cod condition in autumn (i.e. quarter 4), corresponding to the cod main feeding season [38]. Moreover, for autumn long time-series in the population development of the main pelagic fish prey for cod from fishery-independent sources, as well as extent of hypoxic areas, are also available (see below).

Indices of cod abundance (calculated as catch-per-unit-effort, CPUE, no per hour, herein referred to as abundance) from the BITS survey in SDs 25–28 were retrieved from ICES DATRAS database. The indices are based on bottom trawl hauls typically swept between 20 and 100 m depth, whereas hauls shallower than 20 m and deeper than 100 m are very rare [37]. The time-series of cod CPUEs in quarters 1 and 4 are highly correlated, and therefore, in the analyses we used the CPUEs in quarter 1 because of the longer time-series (1991–2014) and the higher number of trawl hauls performed by the survey in this quarter. The CPUE time-series was extended back in time using the relation between CPUEs and cod abundances from the latest accepted stock assessment [39] for the period 1991–2010. The comparison between BITS CPUEs and stock assessment time-series 1991–2010 is shown in electronic supplementary material, figure S1.

Time-series of the biomass of the main pelagic prey of cod (the clupeids herring and sprat), by age and 0.5 cm length-class, in SDs 25–28 were from the autumn Baltic International Acoustic Survey (BIAS, [37]) and historical acoustic surveys from the former Swedish Board of Fisheries (currently the Department of Aquatic Resources, Swedish University of Agricultural Sciences) performed mainly in September–October [14]. Theoretically, not all the sizes of the prey species are equally suitable for all the sizes of cod. We, therefore, also estimated the biomass of the prey sizes most suitable for each length-class of cod by using the size-dependent attack rate (i.e. the rate at which cod of a certain size feed on a certain size of their prey; [40]) as done in [41].

The grey seal data used in this study is based on counts of grey seals hauling out during moult. Monitoring of the grey seal moult has been carried out in the Baltic Sea since the mid-1970s and Swedish surveys have been coordinated by the Swedish Museum of Natural History ever since. In 1989, population trends of Baltic grey seals became part of the Swedish marine monitoring programme with the aim to provide data for analyses of population trends for the Baltic population. Grey seal surveys are carried out during peak moulting time (late May early June during a pre-defined two week period). Even if the surveys are geared to provide a good estimate of population trends for the entire Baltic population, studies of grey seal site fidelity [42,43] have shown that even if grey seals have the potential to move far from their haul-out sites as seen in some studies [44,45] most adult seals seem to show preferences for a certain area, and long-distance movements are less common. This suggests that even if moult counts strictly speaking only refer to the number of seals during a very short period of time during early summer, combined counts for a larger region (e.g. ICES SDs) might be useful to describe population development in this region over time. For our analyses, we, therefore, averaged the seal counts in the sites placed within the SDs 24–28.

Time-series of total areas (km^2^) of hypoxic bottoms (here taken as areas with an oxygen concentration ≤ 1 ml l^−1^, i.e. approx. 1.4 mg l^−1^) by SD (SDs 25–28) were obtained from the Swedish Meteorological and Hydrological Institute (SMHI, www.smhi.se). Time-series of the depth of hypoxic waters (i.e. mean depth at which hypoxia was encountered) by SD were also from SMHI. We used 1 ml l^−1^ as threshold for hypoxia because the Baltic cod has been shown to avoid oxygen concentrations below this value [46]. The time-series of hypoxic areas using the threshold of 1 and 2 ml l^−1^ (the latter more commonly used in the literature as threshold for hypoxia) are highly correlated (*r* = 0.94). We estimated the suitable areas for cod as those with an oxygen concentration > 1 ml l^−1^, excluding the areas shallower than 20 m and deeper than 100 m, as these are not well and consistently sampled during the BITS. Moreover, at depths > 100 m cod is very rare [47]. Consequently, hypoxic areas in the potential distribution area of cod were estimated as those with an oxygen concentration ≤ 1 ml l^−1^ within the 20–100 m depth interval. Hypoxic areas were also used in our study as an indicator of benthic productivity [48,49] and thus of cod benthic feeding opportunities.

To analyse the effect of the different predictors on cod condition we used generalized additive models (GAMs, [50]). The following additive formulation was used:

2.1 $$\text{Condition }=a+s(V_{i})+\cdots +s(V_{n})+\varepsilon ,$$

where *a* is the intercept and *s* the thin plate smoothing spline function [51], *V_i_* … *V_n_* the predictors and ε the random error.

As predictors for cod condition (response) the following variables were employed in the full models: cod abundance (i.e. density-dependence), biomass of herring and sprat (as total biomass or as biomass of the prey sizes most suitable for each length-class of cod) included both separately or taken together as clupeids (i.e. food availability), seal abundance (proxy for infection risk from seal parasites) and extent of hypoxic areas (potentially acting directly and indirectly on cod condition, see Introduction and Discussion). These predictors were selected based on acknowledged ecological and physiological mechanisms potentially affecting cod condition [19,21,25,33]. Hypoxic areas, for instance, can have an indirect effect on cod condition by reducing the suitable distribution area, which can induce crowding and potentially density-depend effects [52]. To investigate this hypothesis, in all the final models that included cod abundance, the factor ‘cod abundance’ was replaced by ‘cod density’, estimated as the ratio cod abundance/suitable area. In these models, we eliminated the predictor ‘hypoxic areas’ in order not to use the same single predictor twice in the models. Cod abundance in number, rather than biomass, was used in the GAMs because biomass contains already by definition a signal related to body weight, making response (condition) and predictor (biomass) not independent *a priori*. All variables were expressed as standardized anomalies prior to analysis (*X′* = *X* − mean/s.d.).

To find the best possible set of predictors, we ran a model selection based on statistical significance and generalized cross validation (GCV) using a backward stepwise procedure. The GCV criterion allows an optimal trade-off between the amount of deviance explained by the model and the model complexity measured through the equivalent degrees of freedom. From the full model, the predictor with the lowest *p*-value was excluded at each step and the model was run again, until the GCV reached a minimum. If excluding a predictor increased the GCV, the predictor was retained and the procedure ended. We limited the maximum degrees of freedom acceptable for each term to *k* = 4. A normal distribution with an identity function was used in the GAMs. We calculated the deviance explained by the final models, and the deviance contribution of each predictor based on the percentage difference in explained deviance of the final models after deletion of one predictor at a time while keeping the others (i.e. with replacement) (Difference Dev. Expl. %) [15]. Residuals were inspected for deviation from the assumption of normality and no autocorrelation using graphical methods [53]. We modelled the temporal changes in cod condition in two separated time periods, 1976–1993 and 1994–2014. The early 1990s have been characterized by a shift in the structure [15,54] and functioning [55] of the Central Baltic Sea ecosystem. Therefore, we attempted to investigate whether the variations in cod condition could be attributed to different ecological processes before and after the shift. A model in which the whole time period was analysed was also attempted.

The statistical analyses were performed using the mgcv library of R v. 3.0.2 (www.r-project.org) and Statistica v. 6.1. The significance level was set to *α* = 0.05 for all tests.

## 3. Results

### 3.1. Spatio-temporal patterns in cod condition

**Figure 2.**
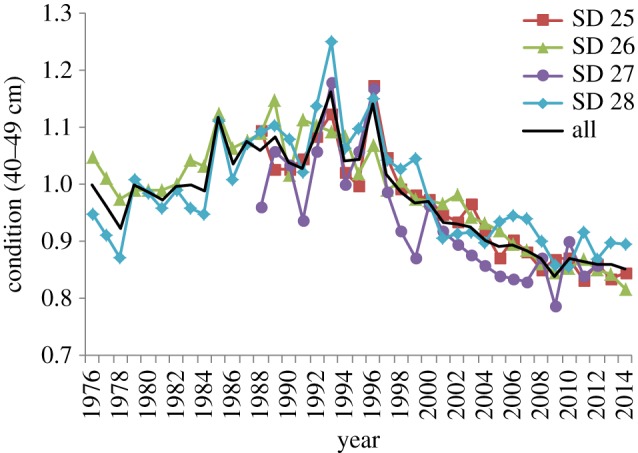
Temporal developments of mean cod condition in the different subdivisions (SDs) of the Central Baltic Sea for cod 40–49 cm. The black thick line is the average between the SDs.

**Figure 3.**
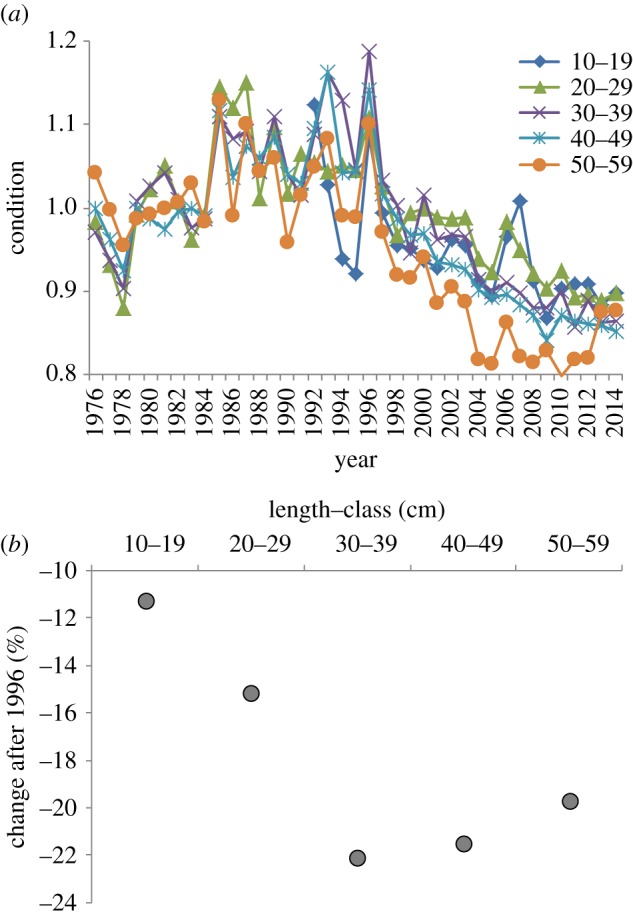
(*a*) Temporal developments of mean cod condition in the Central Baltic Sea (average SDs 25–28) for different size-classes; (*b*) changes in condition (%) between 1992–1996 and 2010–2014.

**Figure 4.**
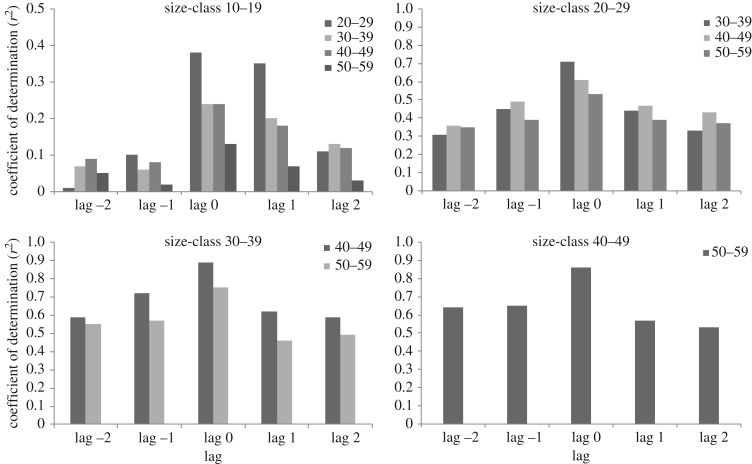
Coefficients of determination of the relation between the time-series of conditions of the different cod size-classes, at different time lags. The condition of each length-class was related to the condition of the other length-classes at +2, +1, −1 and −2 time (year) lags.

The condition of cod in the Central Baltic Sea (SDs 25–28) increased between 1976 and 1995, whereas it dropped from the mid-1990s to 2010, and stabilized at a low level in the last few years in all the SDs and size-classes (figures 2 and 3*a*; electronic supplementary material, figure S2). The drop in cod condition after the mid-1990s was strongest for the intermediate and larger cod (length-classes 30–39 to 50–59 cm, that decreased approximately 20–22%) compared with the 10–19 cm length-class (11% decrease) and the 20–29 cm length-class (15% decrease) (figure 3*a,b*). However, the temporal variations in condition were synchronous for all the length-classes, as shown by pairwise correlations at different time lags (figure 4). The condition of cod in the length-class 40–49 cm was used as response variable in the GAM modelling.

### 3.2. Cod abundance, clupeid biomass and seals

**Figure 5.**
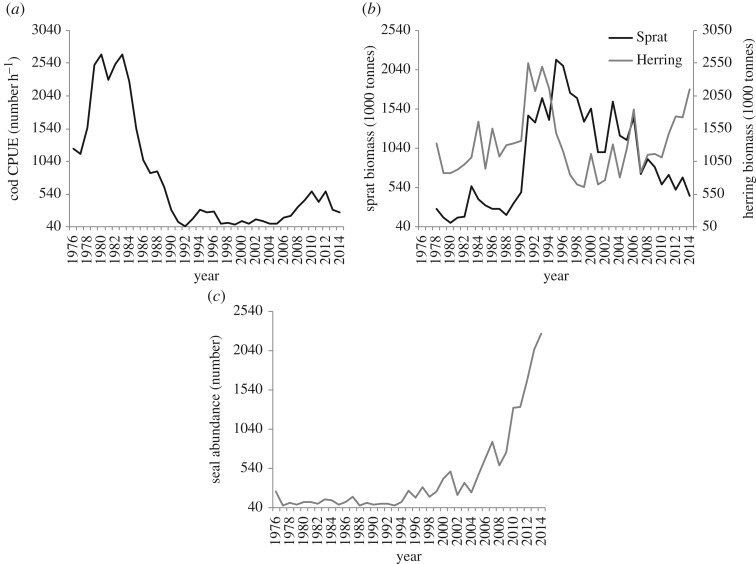
Time-series of (*a*) cod abundance, (*b*) sprat and herring biomass and (*c*) seal abundance, used as predictors to explain cod condition in the GAMs.

Cod adult abundance (length ≥ 30 cm, hereafter referred to as cod abundance) increased from the mid-1970s to the mid-1980s, followed by a drop that culminated in the early 1990s. Cod abundance has been low from 1991 to the mid-2000s. Afterwards, it increased up to the late 2000s, followed by a slight decrease (figure 5*a*). The abundance of cod ≥ 30 cm was used as predictor in the GAM modelling because they constitute the spawning fraction of the Baltic cod population [17] and compete for common resources [40]. In the area of main cod distribution (SDs 25–28), sprat biomass was low until the early 1990s, increased rapidly up to the mid-1990s and thereafter decreased again to the levels of the start of the time-series. Herring biomass showed an increase up to the early 1990s, followed by a decrease up to the early 2000s and a further increase in the last years of the time-series (figure 5*b*). Seal abundance was low up to the early 1990s, a little higher between 1993 and 2003, and since the mid-2000s it has increased strongly (figure 5*c*).

### 3.3. Hypoxic areas and areas suitable for cod

**Figure 6.**
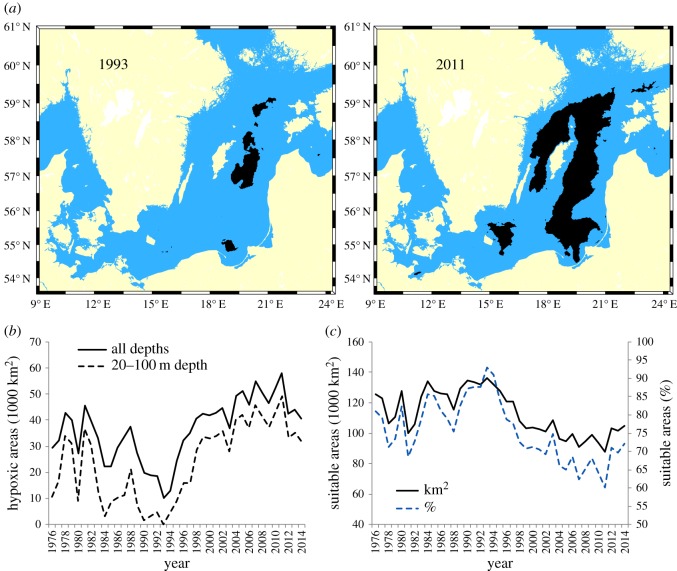
(*a*) Maps of hypoxic areas (less than or equal to 1 ml l^−1^ oxygen concentration) in 1993 and 2011; (*b*) time-series of total hypoxic areas (all depths), and hypoxic areas between 20 and 100 m depth, the latter used as predictors to explain cod condition in the GAMs; (*c*) time-series of suitable areas for cod (more than 1 ml l^−1^ oxygen concentration) between 20 and 100 m depth, in absolute values and in percentage. The time-series refer to the Central Baltic Sea (SDs 25–28).

The changes in the extent of hypoxic areas (less than or equal to 1 ml l^−1^ oxygen concentration) between 1993 (lowest level in the time period considered) and 2011 (highest level in the time period considered) are shown as an example in the maps of figure 6*a*. Hypoxic areas in SDs 25–28 decreased from the late 1970s to the lowest levels in mid-1990s (approx. 10 × 10^3^ km^2^), increased strongly up to the mid-2000s, and remained constant afterwards at around 50 × 10^3^ km^2^ (figure 6*b*). Hypoxic areas within the main depth interval of cod (i.e. down to 100 m depth) showed very similar patterns (figure 6*b*). Suitable areas for cod (areas with oxygen concentration > 1 ml l^−1^ excluding depths shallower than 20 m and deeper than 100 m) increased from the late 1970s to the mid-1990s (approx. 140 × 10^3 ^km^2^, corresponding to 90% of the total area), decreased until the late 2000s and remained thereafter stable at around 90 × 10^3^ km^2^ (corresponding to 65% of the total area) (figure 6*c*). In percentage, the decrease in suitable areas between the early 1990s and the late 2000s has been approximately 30%.

### 3.4. Modelling cod condition

**Figure 7.**
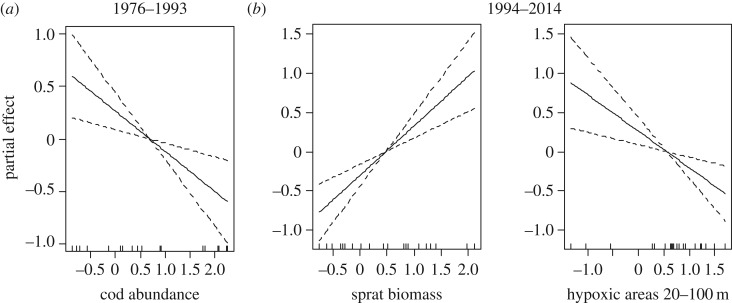
Results of the GAMs (final models) for the two separated time periods (1976–1993 and 1994–2014). The partial effects of each predictor on cod condition are shown. See table 1 for details on the statistics.

The final model of cod condition for the period 1976–1993 explained 36.4% of the total deviance (table 1). Cod abundance was the only predictor of cod condition (negative effect) retained by the selection procedure (figure 7*a*). Cod abundance, sprat and herring biomass and seal abundance were discarded by the model selection procedure (their stepwise elimination decreased the GCV of the model). The residuals did not violate the normality and homogeneity assumptions, and were not autocorrelated (electronic supplementary material, figure S3). The use of ‘cod density’ instead of ‘cod abundance’ improved the overall performance of the model (38.9% of the deviance explained and lower GCV; electronic supplementary material, table S1).

**Table 1.** Results of the GAMs (final models) for the two separated time periods (1976–1993 and 1994–2014). The generalized cross validation (GCV), the deviance explained (Dev. Expl.) and the number of observations (*n*) are indicated. For each predictor, the degrees of freedom (d.f.), the significance value (*p*) and the deviance explained by the model excluding the corresponding predictor (Difference Dev. Expl. %) are provided. Predictors without statistics indicate that they were excluded by the backward stepwise model selection.

GAMs	predictors	GCV	*r*^2^ (adj.)	Dev. Expl. (%)	*n*	d.f.	*F*	*p-*value	Difference Dev. Expl. (%)	single predictor
model 1976–1993	cod abundance					1.00	9.17	0.008	—	36.4
	sprat biomass									
	herring biomass									
	seal abundance									
	hypoxic areas 20–100 m									
	final model	0.376	0.33	36.40	18					
model 1994–2014	cod abundance									
	sprat biomass					1.00	18.26	0.0004	20.87	75.50
	herring biomass									
	seal abundance									
	hypoxic areas 20–100 m					1.00	9.19	0.007	8.93	65.60
	final model	0.187	0.81	82.90	21					

The final model for the period 1994–2014 explained 82.9% of the total deviance (table 1). Sprat biomass (positive effect) and hypoxic areas (negative effect) were the predictors of cod condition retained in the final model (figure 7*b*). Cod abundance, herring biomass and seal abundance were discarded by the model selection procedure (their stepwise elimination decreased the GCV of the model). The residuals did not violate the normality and homogeneity assumptions and were not autocorrelated (electronic supplementary material, figure S4). The use of the ‘biomass of the most suitable prey size’ instead of ‘prey biomass’ increased the deviance explained by the model (87.2%) but also increased its GCV (electronic supplementary material, table S2).

**Figure 8.**
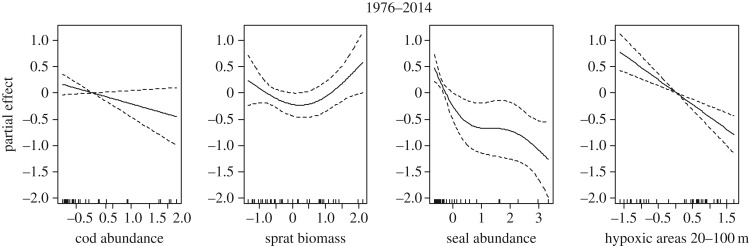
Results of the GAM (final model) for the whole time-series (1976–2014). The partial effects of each predictor on cod condition are shown. See table 2 for details on the statistics.

We also modelled the temporal changes in cod condition in the whole time period (1976–2014). The final model explained 85.9% of the total deviance (table 2). Cod abundance (negative effect), sprat biomass (positive effect), seal abundance (negative effect) and hypoxic areas (negative effect) were the predictors of cod condition retained in the final model (table 2 and figure 8). The residuals did not violate the normality and homogeneity assumptions, and were not autocorrelated (electronic supplementary material, figure S5). The use of the ‘biomass of the most suitable prey size’ instead of ‘prey biomass’ decreased the deviance explained by the model (84.6%) and increased its GCV (electronic supplementary material, table S3). We also ran two models in which we excluded the term ‘hypoxic areas’ and compared the statistics of the models using ‘cod abundance’ or ‘cod density’ as density-dependent factor. The use of ‘cod density’ instead of ‘cod abundance’ improved the overall performance of the model, i.e. had a higher explained deviance (79.6% versus 78.4%) and lower GCV. Moreover, the density-dependent term ‘cod density’ became significant (electronic supplementary material, table S4).

**Table 2.** Results of the GAM (final model) for the whole time-series (1976–2014). The generalized cross validation (GCV), the deviance explained (Dev. Expl.) and the number of observations (*n*) are indicated. For each predictor, the degrees of freedom (d.f.), the significance value (*p*) and the deviance explained by the model excluding the corresponding predictor (Difference Dev. Expl. %) are provided. Predictors without statistics indicate that they were excluded by the backward stepwise model selection.

GAMs	predictors	GCV	*r*^2^ (adj.)	Dev. Expl. (%)	*n*	d.f.	*F*	*p*-value	Difference Dev. Expl. (%)	single predictor
model 1976–2014	cod abundance					1.00	2.71	0.11	2.33	2.03
	sprat biomass					2.03	3.12	0.049	7.10	39.40
	herring biomass									
	seal abundance					2.57	7.68	0.0007	10.48	63.00
	hypoxic areas 20–100 m					1.00	19.77	0.0001	8.73	67.20
	final model	0.229	0.83	85.90	37					

## 4. Discussion

Our study suggests that the temporal variations in the condition of the Eastern Baltic cod have been caused by multiple biotic and abiotic factors. Our analyses, in fact, show a strong negative correlation between cod condition and the extent of hypoxic areas and a positive correlation between cod condition and the biomass of sprat after the mid-1990s. On the other hand, a negative relation between cod condition and cod abundance, as indication of intra-specific competition, was evident before the mid-1990s in correspondence of large variations in cod population abundance.

The factors affecting the condition of other gadoid populations, including cod, have been intensively investigated worldwide. These factors range from physical forcing and prey availability to selective fishing and internal population control such as intra-specific density-dependence [3,21,56,57]. The Baltic Sea has undergone drastic spatio-temporal changes during the past 40 years, both in the biological and abiotic components [15,54,58], which can have affected the condition of the cod. In the following paragraphs, we discuss the recent development of cod condition in the Baltic Sea in view of the main ecosystem changes that were accounted for in our analyses.

### 4.1. Food availability: density-dependence and pelagic fish prey shortage

One of the main biotic changes in the Baltic Sea ecosystem during the past four decades has been the large increase in the total population size (SDs 22-23) of sprat (i.e. the main pelagic fish prey for cod) starting in the early 1990s. After having reached a peak in the mid-1990s, the sprat population size somewhat decreased again but has remained at relatively high levels since the early 2000s [17]. Beside the changes in total population size, there has been a spatial reallocation of the populations of sprat, from being uniformly distributed in the Baltic Sea to being concentrated in the northeastern areas [14,19]. This has ultimately resulted in an increase in sprat abundances in the northern areas (SDs 29 and 32) but a reduction in the Central Baltic Sea (SDs 25–28) where cod has been concentrated during the past 25 years [19,59]. The strong positive correlation between cod condition and sprat biomass found in our statistical analyses for the period 1994–2014 reflects, therefore, the decrease in prey biomass in the current area of cod distribution and the increased spatial mismatch between cod and its main pelagic fish prey. These results conform to Eero *et al.* [21] who found for the Bornholm Basin (SD 25) a positive relation between the condition of old cod individuals landed by the fishery and the ratio ‘pelagic prey/cod abundance’ suggesting that cod condition was driven by *per capita* food availability. Our analyses performed separately for the two periods, 1976–1993 and 1994–2014, showed evidence for density-dependence in the first period and food limitation in the second period. However, while in the second period with low cod abundance the sprat dynamics have been mainly driven by recruitment variations, in the first period it was driven by cod predation [55,60]. Therefore, it is likely that in the first period the fast collapse of the cod population and the subsequent predation release on its pelagic prey has resulted in the increase in cod condition observed up to the early 1990s. In fact, the use of sprat biomass, instead of cod abundance, in the final GAM model for the period 1976–1993 also evidenced a positive and significant relationship with cod condition (29.3% Dev. Expl., *p* = 0.03). These results suggest that feeding opportunities on pelagic prey, either driven by density-dependence or food limitation, have been important to shape cod condition in the past 40 years.

### 4.2. Hypoxic areas: physiology, benthic food and density-dependence

**Figure 9.**
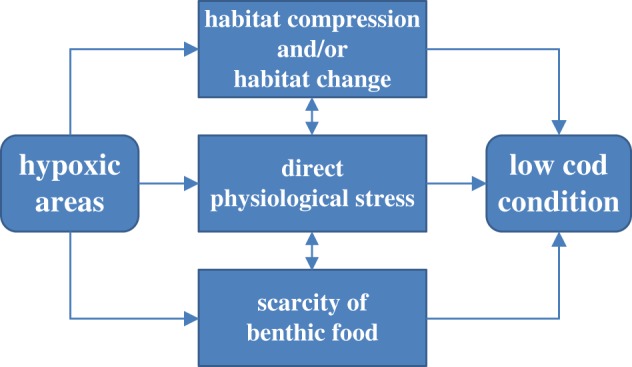
Schematic representation of the mechanisms potentially explaining the negative relationship between hypoxic areas and cod condition. See the text for a detailed explanation of each mechanism.

Although feeding opportunities on pelagic fish prey were important to explain cod condition, another strong factor in our model was represented by the extent of low-oxygen bottoms. The effect of hypoxic areas was especially strong in our models in the second time period, i.e. 1994–2014. Hypoxic areas increased dramatically during the 1990s, levelling off since the mid-2000s, which depicts an inverse pattern to that shown by cod condition. Hypoxic areas can affect Baltic cod through several mechanisms, which are not mutually exclusive and whose effects may, therefore, sum up to lead to the observed changes in condition (figure 9).

Firstly, adverse oxygen circumstances can result in physiological and behavioural stress in fish. Experimental studies have shown a decline in fish growth (in terms of increase in body size or in body condition) when fish were exposed to hypoxia ([26,30,33] and references therein) which can be explained by an increase in energetic costs for ventilation which decreases the amount of energy available for growth [61] and/or by a decrease in food intake [30,62,63] as observed also in cod [33,64]. This would allow fish to preserve energy, and therefore, reduce total oxygen demand [30]. Other experimental studies have shown that cod kept at low oxygen may rely on energetically expensive anaerobic energy production [65]. Other experiments found that fish (including cod) swimming activity and speed is reduced by hypoxia ([32] and references therein), probably as an adaptive response to offset major metabolic stress and, therefore, for survival [31], which may also affect food intake in the wild. Previous analyses on Baltic cod stomach data for the period 2007–2014 showed that the frequency of occurrence of both pelagic and benthic prey in the stomachs was lower in regions with prevalence of hypoxic bottoms, whereas the frequency of occurrence of empty stomachs increased in these regions [66], providing support for a direct effect of experienced hypoxia on food intake.

This first mechanism (the physiological mechanism) that can be advocated to lay behind the strong relation between cod condition and hypoxic areas found in our statistical models presupposes that cod do not completely avoid low-oxygen areas. In [67], using tagged Baltic cod individuals, it was shown that cod visit hypoxic waters, briefly but frequently, probably in search of benthic preys [65], which generally are more tolerant to hypoxia than fish [68], providing support for the occurrence of this mechanism. According to this interpretation, as most fish avoid oxygen concentrations that would decrease their growth [25], in our analyses 1 ml l^−1^ could be considered as a sub-lethal threshold that cod tend to avoid when not feeding in deeper layers with an oxygen concentration even lower. However, laboratory experiments on Atlantic cod from Canada showed a significant decrease in condition already at 3 ml l^−1^ [33] which corresponds to the median sub-lethal threshold found for fish in a meta-analysis by Vaquer-Sunyer & Duarte [68]. Moreover, laboratory experiments performed on Atlantic cod showed that 50% mortality (lethal oxygen threshold) was reached at a dissolved oxygen of around 0.5–1.3 mg l^−1^ (corresponding to 0.2–1 ml l^−1^) at a temperature around 5–10°C [69], which is the average autumn temperature in the southern Baltic Sea just above the halocline. The oxygen concentration of 1 ml l^−1^ used in our study as avoidance level (according to [46]) could, therefore, be the level that cod escape for survival. In this case, dwelling at an oxygen concentration just above 1 ml l^−1^ would still impair condition. It could, however, be that Baltic cod is adapted to low-oxygen environment and has, therefore, lower oxygen thresholds than other cod populations, but experimental studies relating mortality and oxygen concentration for Baltic cod are currently lacking.

A second mechanism (benthos productivity mechanism) we propose to explain the negative relation between cod condition and hypoxic areas is the availability of the benthos, an important component of the diet across all sizes of cod [20]. Benthic productivity is highly affected by low-oxygen concentrations in many coastal, shelf and estuarine areas worldwide [24]. As in most of these areas, in the Baltic Sea the decrease in oxygen level in deep waters and the increase in the extent of hypoxic and anoxic areas have reduced the benthic biodiversity and the overall benthic productivity via habitat loss [48,49]. This has resulted in the elimination of benthic macrofauna over vast areas, degradation of benthic communities and disruption of benthic food webs in deep waters [48,70]. One of the main benthic prey for large cod is the burrowing isopod *Saduria entomon* [20]. This species is highly tolerant to low-oxygen concentrations and has a mechanism to detoxify sulfides [71,72], and it is, therefore, supposed to cope better with a decrease in oxygen. It could, therefore, be that the spatial overlap between cod and *Saduria entomon* has diminished in the past two decades due to the increasing extent of hypoxic areas, constituting an additional factor explaining the decrease in cod condition (see also the ‘habitat compression mechanism’ below). Previous analyses on Baltic cod stomach data for the period 2007–2014 showed that the proportion of cod with benthic food in their stomachs decreased in regions with prevalence of hypoxic bottoms [66]. This can be explained by a reduction of the availability of benthic prey, and also by a change in cod behaviour that in situations of deep-water hypoxia become more pelagic [46] probably feeding proportionally more on pelagic prey.

A third mechanism (habitat compression mechanism) we propose mediating the link between hypoxia and cod condition is represented by the habitat compression that hypoxic areas may cause on aquatic populations, with several examples spanning worldwide from open oceans, coral reefs and estuaries [25,27,29,73–75]. The Baltic cod avoid oxygen below 1 ml l^−1^ [46] and our estimations revealed that the areas suitable for cod (i.e. areas with oxygen > 1 ml l^−1^) has decreased by around 30% from the early 1990s. The spatial compression of the suitable habitat and the consequent crowding of the population may trigger density-dependent processes, such as a decline in condition. In the case of the Baltic cod, the habitat compression has been concomitant with an increase in cod population abundance since the mid-2000s, potentially worsening the density-dependent response. Our analyses showed that cod density (determined as the ratio between cod abundance and suitable areas, i.e. water with oxygen > 1 ml l^−1^) was a better explanatory variable than cod abundance, suggesting that crowding, mediated by the shrinkage of suitable habitats, has been an important factor in the decrease of cod condition in the past 20 years (see [29,52], for an example, from the Neuse River Estuary in USA). This conclusion is supported also by the fact that the cod suffering the strongest decrease in condition were the large ones that dwell deeper [47], and therefore, are more prone to changes in the extent of hypoxic areas.

### 4.3. Seal parasites

Seal parasites have been advocated as a factor that could have contributed to the decrease in cod condition during the past three decades [18,19]. During recent years the infestation by two different parasites, the cod worm residing in the cod flesh and the liver worm residing in the cod liver, has increased [18,34,35]. Seals are the final host in which the parasites produce their eggs [76], and therefore, it could be supposed that with an increasing grey seal population [77], the risk of cod infection would also augment [18,36,78]. In our models, seal abundance was the first variable eliminated by the model selection procedure when the analyses were conducted separately for the two time periods. On the other hand, in the model using the whole time-series seal abundance remained as an important explanatory factor in the final model. However, although the seal population started to increase already from the mid-1990s, its exponential increase started after the early 2000s and is still continuing, whereas cod condition started to drop already in the mid-1990s and has levelled off since 2010. This suggests that, although an effect of seal parasites on heavily infected cod individuals cannot be excluded, this effect could be minor at the population level in comparison with the other factors.

## 5. Conclusion

The decrease in the condition of Baltic cod that started in the mid-1990s has been a matter heavily debated in the past few years both in the scientific forums and media, as it has large economic and ecological implications [16,18,19]. Our study is the first trying to disentangle the importance of potential multiple biotic and abiotic factors contemporarily acting on the condition of cod in the Baltic Sea, which could help the management of cod fisheries and improve the health of the ecosystem as a whole. We have shown that the drastic variations in cod condition during the past 40 years are associated with a combination of an increased extent of hypoxic areas, density-dependence and pelagic fish prey (sprat) availability. The literature and the new statistical analyses performed in our study suggest that the link between hypoxic areas and cod condition can be mediated by metabolism [33], lower food intake [66], reduced abundance of benthic fauna [48,49] and habitat compression probably inducing density-dependence ([52] and this study). In future studies, effort should be made to investigate the relation between cod condition, food availability (both pelagic and benthic prey) and stomach contents [20,66], to better understand the physiological and ecological mechanisms leading to actual food intake and diet composition.

From a management perspective, our results highlight the importance of regulating anthropogenic nutrient inputs, to dampen eutrophication and thus combat oxygen depletion [79] also for fish populations and the future of the fisheries in the Baltic Sea. Moreover, the strong effect of pelagic prey biomass on cod condition found in our study supports the recent ICES Advice to limit the fishery after pelagic fish in the current main distribution area of cod [80] to preserve important food resources for cod. Experimental studies have evidenced that cod with low condition have a higher chance to die because of starvation [6], and therefore, a negative effect on cod survival could also be expected for the Baltic Sea [81]. Our results evidence the necessity to link tightly environmental and fisheries management issues to assure the health of fish populations and the profitability of the fishery.

## Supplementary Material

Comparison between stock assessment and survey CPUEs, additional figures on cod condition and GAMs statistics.

## Data accessibility

The data are available on the Dryad Digital Repository http://dx.doi.org/10.5061/dryad.875fb [82].
