# Supplementary material for: Hypoxic areas, density-dependence and food limitation drive the body condition of a heavily exploited marine fish predator
Source: R Soc Open Sci. 2016 Oct 26;3(10):160416. doi: 10.1098/rsos.160416 (PMC5098982; doi:10.1098/rsos.160416)
Supplement: Comparison between stock assessment and survey CPUEs, additional figures on cod condition and GAMs statistics. [file rsos160416supp1.docx]

**Electronic Supplementary Material**

**Hypoxic areas, density-dependence and food limitation drive the body condition of a heavily exploited marine fish predator**

Michele Casini*, Filip Käll, Martin Hansson, Maris Plikshs, Tatjana Baranova, Olle Karlsson, Karl Lundström, Stefan Neuenfeldt, Anna Gårdmark, Joakim Hjelm

Figure S1. (a) Comparison between cod abundance estimation from stock assessment (fish ≥ age 3) and CPUE from BITS survey (fish ≥ 30 cm). (b) Relation between the cod abundance estimation from stock assessment (fish ≥ age 3) and CPUE from BITS survey (fish ≥ 30 cm) between 1991-2010. The relation was used to prolong the CPUE time-series back in time.

Figure S2. Temporal developments of cod mean condition in the different Subdivisions (SDs) of the Central Baltic Sea for different size-classes. SD 28 is not shown for the size-class 10-19 cm due to the low sample size.

Figure S3. Analysis of the residuals of the GAM (final model) for the period 1976-1993. *ACF*: autocorrelation function.

Figure S4. Analysis of the residuals of the GAM (final model) for the period 1994-2014. *ACF*: autocorrelation function.

Figure S5. Analysis of the residuals of the GAM (final model) for the period 1976-2014. *ACF*: autocorrelation function.

Table S1. Results of the GAM for the period 1976-1993 in which “cod density” was used instead of “cod abundance”**.** The Generalized cross validation (GCV), the deviance explained (Dev. Expl.) and the number of observations (n) are indicated. For each predictor, the degrees of freedom (df), the significance value (P) and the deviance explained by the model excluding the corresponding predictor (Difference Dev. Expl. %) are provided. Predictors without statistics indicate that they were excluded by the backward stepwise model selection.

Table S2. Results of the GAM for the period 1994-2014 in which the “biomass of the most suitable prey size, SS” was used instead of “prey biomass”**.** The Generalized cross validation (GCV), the deviance explained (Dev. Expl.) and the number of observations (n) are indicated. For each predictor, the degrees of freedom (df), the significance value (P) and the deviance explained by the model excluding the corresponding predictor (Difference Dev. Expl. %) are provided. Predictors without statistics indicate that they were excluded by the backward stepwise model selection.

Table S3. Results of the GAM for the period 1976-2014 in which the “biomass of the most suitable prey size, SS” was used instead of “prey biomass”**.** The Generalized cross validation (GCV), the deviance explained (Dev. Expl.) and the number of observations (n) are indicated. For each predictor, the degrees of freedom (df), the significance value (P) and the deviance explained by the model excluding the corresponding predictor (Difference Dev. Expl. %) are provided. Predictors without statistics indicate that they were excluded by the backward stepwise model selection.

Table S4. Results of the GAM for the period 1976-2014 in which “cod abundance” or “cod density” was used as density-dependent factor. In this model hypoxic areas were excluded, since oxygen information is already included in the estimation of cod density. The Generalized cross validation (GCV), the deviance explained (Dev. Expl.) and the number of observations (n) are indicated. For each predictor, the degrees of freedom (df), the significance value (P) and the deviance explained by the model excluding the corresponding predictor (Difference Dev. Expl. %) are provided. Predictors without statistics indicate that they were excluded by the backward stepwise model selection.
